# Supplementary material for: Highly Sensitive Fluorescent Detection of Acetylcholine Based on the Enhanced Peroxidase-Like Activity of Histidine Coated Magnetic Nanoparticles
Source: Nanomaterials (Basel). 2021 May 1;11(5):1207. doi: 10.3390/nano11051207 (PMC8147487; doi:10.3390/nano11051207)
Supplement: Supplementary file 1 [file nanomaterials-11-01207-s001.zip › nanomaterials-1188677-supplementary.pdf]

nanomaterials

Supplementary Materials

Highly sensitive fluorescent detection of acetylcholine based on the enhanced peroxidase-like activity of histidine coated magnetic nanoparticles

Hong Jae Cheon,† Quynh Huong Nguyen,† and Moon Il Kim*

Department of BioNano Technology, Gachon University, 1342 Seongnamdae-ro, Sujeong-gu, Seongnam, Gyeonggi 13120, Republic of Korea; hjchun1201@naver.com (H.J.C.); pruedence122@gmail.com (Q.H.N.)
***Correspondence: moonil@gachon.ac.kr (M.I.K.); Tel.: +82-31-750-8563**

Methods for investigating the mechanism for the peroxidase-like activity of His@MNPs

Generation of the free ·OH radicals as the reactive intermediates during the decomposition of H_2O_2 mediated by the peroxidase activity of His@MNPs was demonstrated via a fluorescence assay. To perform this experiment, terephthalic acid (TA) probe was deployed since it can react with ·OH radicals to generate highly fluorescent 2-hydroxyterephthalic acid that exhibits a signature emission at around 430 nm. The experiment was conducted in a mixture containing 100 µL TA (5 mM), 100 µL sample (100 µg/mL pristine MNPs or His@MNPs) and 100 µL H_2O_2 (100 mM) in a 700 µL of 0.1 M sodium acetate buffer (pH 5.5). The mixture then was incubated in dark condition for 12 hours. Consequently, the fluorescence spectra were recorded at the excitation wavelength at 315 nm, in a black 96 well-plate using microplate reader.

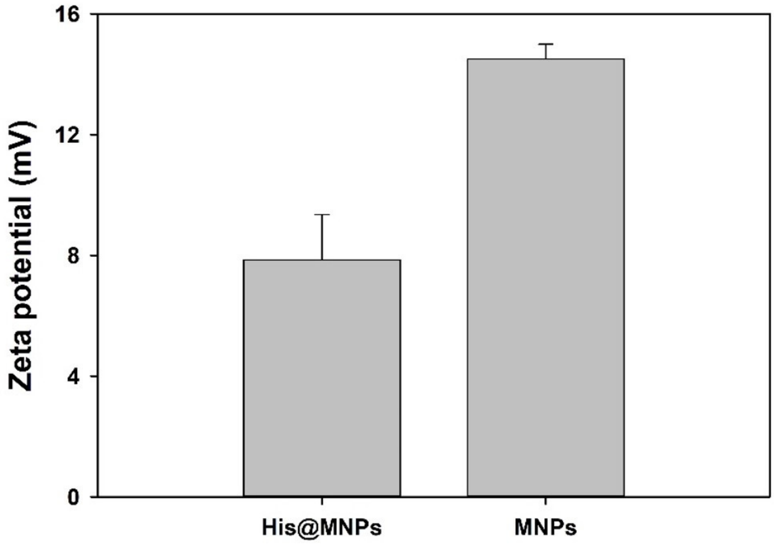

Figure S1. Zeta potential values of His@MNPs and pristine MNPs in sodium acetate buffer (0.1M, pH 4.0).

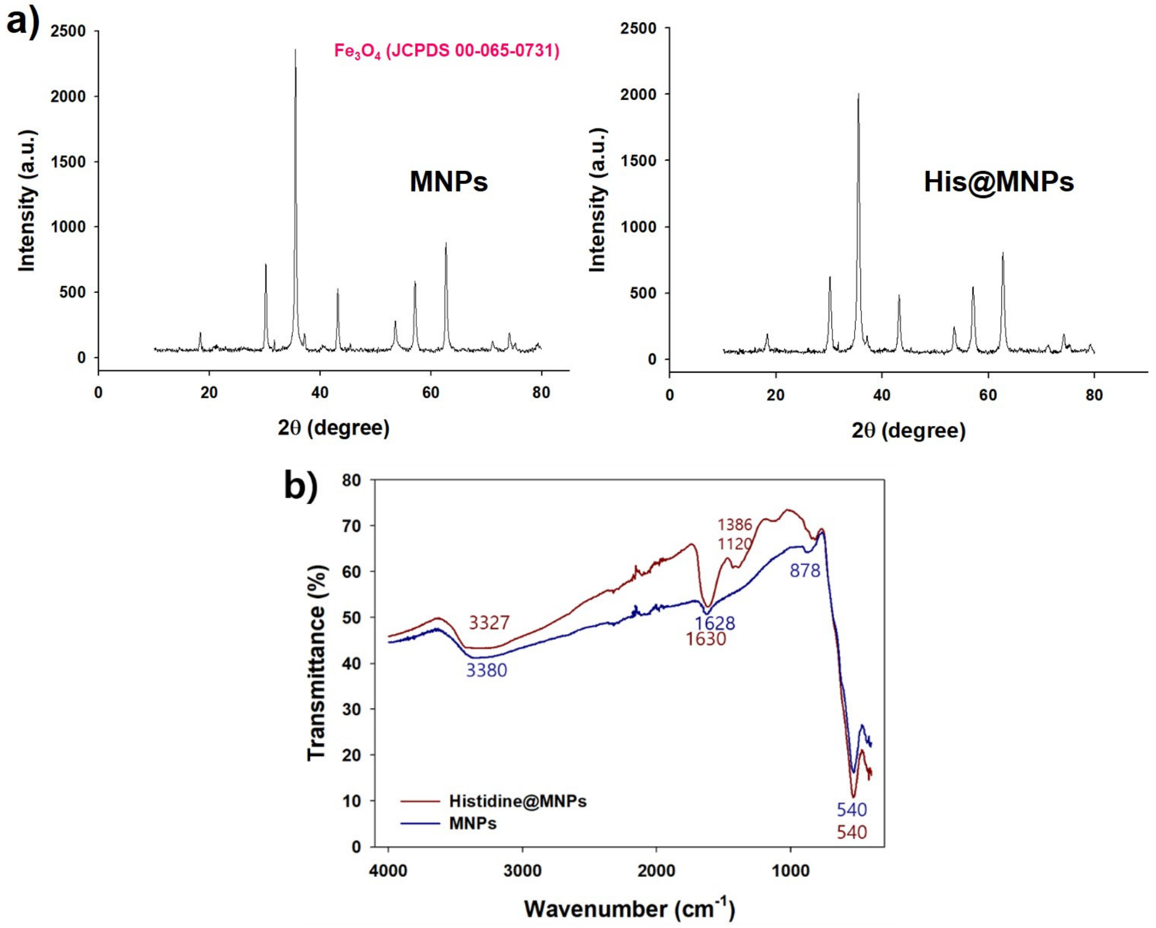

Figure S2. a) XRD and b) FT-IR spectra of His@MNPs and pristine MNPs.

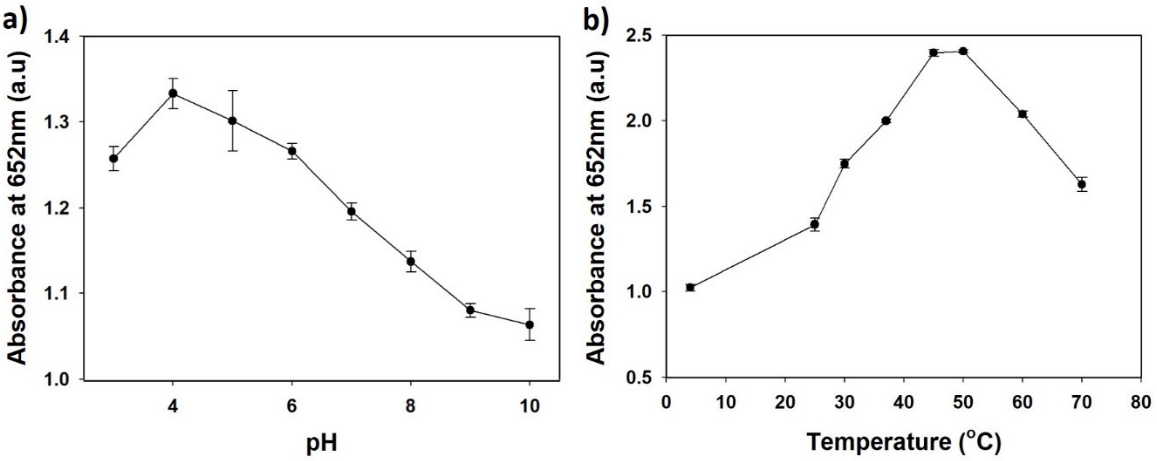

Figure S3. Effects of a) pH and b) temperature on the catalytic activity for TMB oxidation catalyzed by His@MNPs.

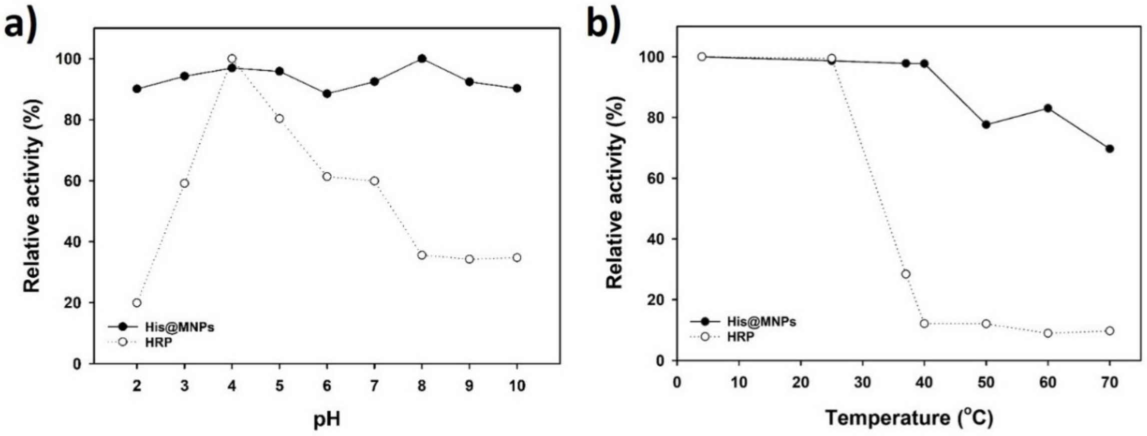

Figure S4. Catalytic stabilities of His@MNPs and HRP against a) pH and b) temperature. In this experiment, His@MNPs and HRP were incubated at diverse a) pH and b) temperature conditions for 5 h and then their peroxidase activities were measured at a standard assay conditions (pH 4.0). The maximum relative activity in the y-axis of each curve was set at 100%.

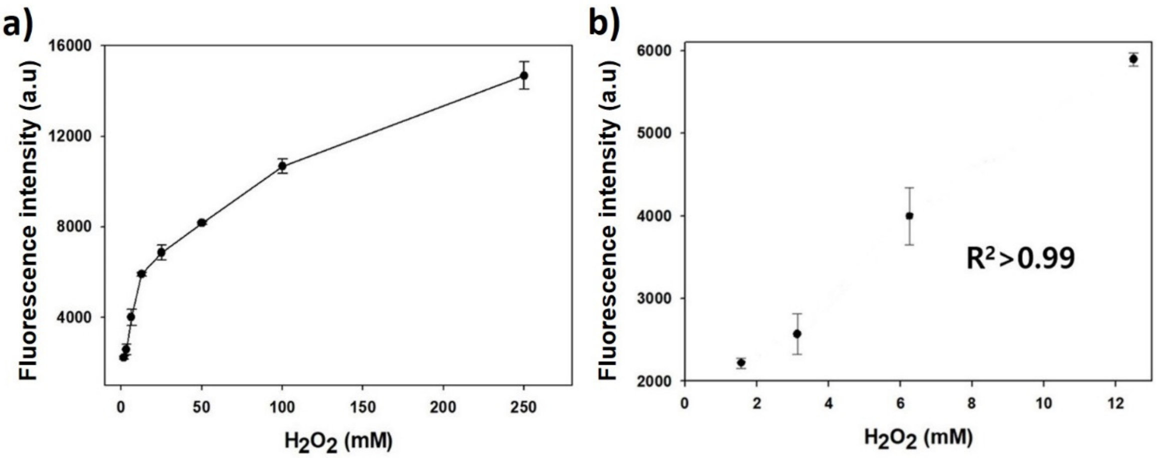

Figure S5. a) Dose-response curve for the detection of H_2O_2 and b) their corresponding linear calibration plot using His@MNPs as peroxidase mimics and AUR substrate.

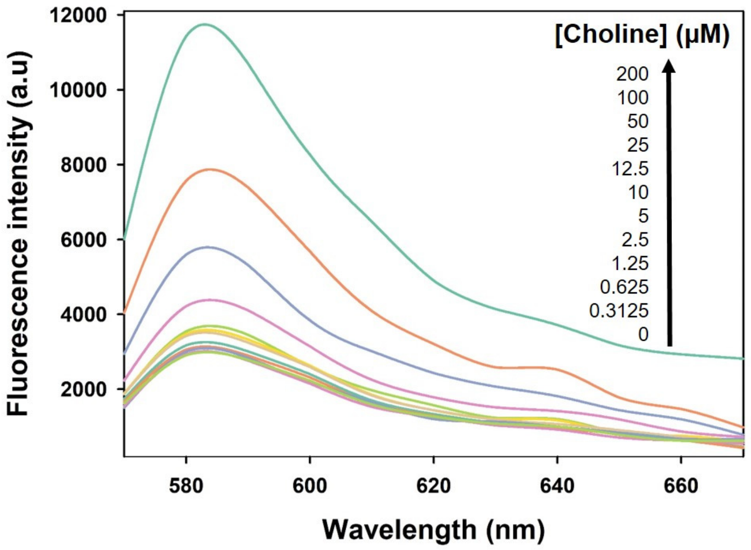

Figure S6. Fluorescence emission spectra for the detection of choline.

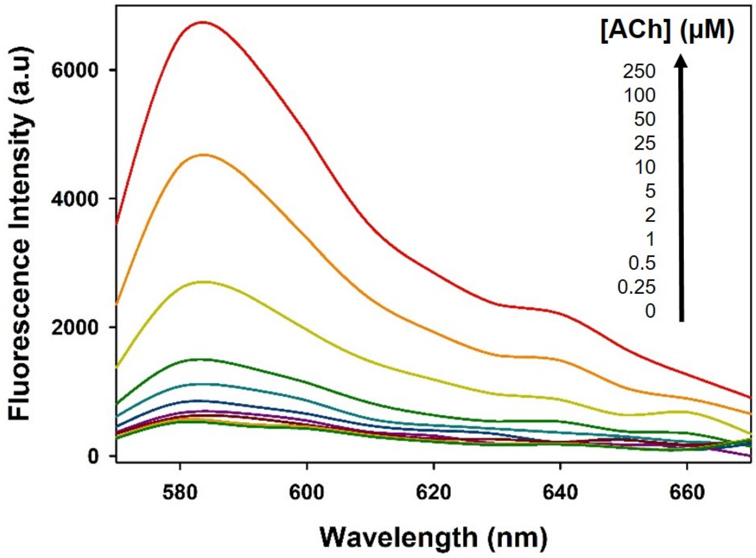

Figure S7. Fluorescence emission spectra for the detection of ACh.

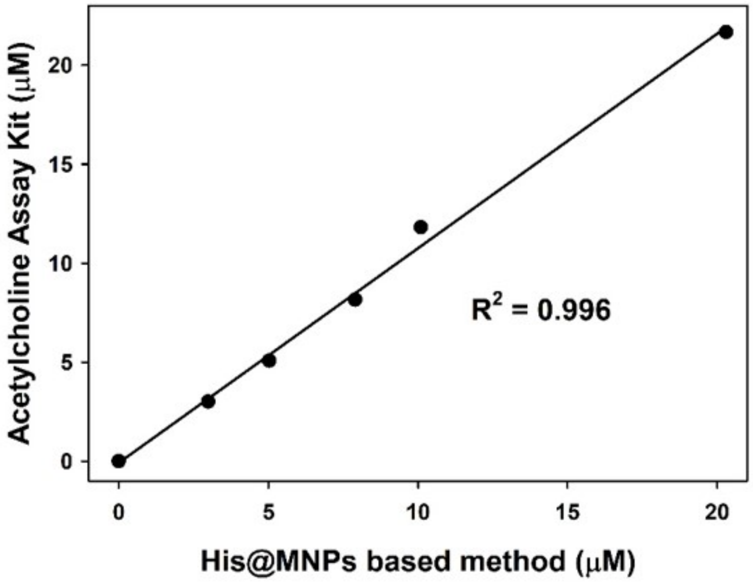

Figure S8. Correlation between His@MNPs based method (x) and Choline/Acetylcholine detection kit (y) for the quantitative determination of ACh.

© 2021 by the authors. Submitted for possible open access publication under the terms and conditions of the Creative Commons Attribution (CC BY) license (http://creativecommons.org/licenses/by/4.0/).
